# Mantle Cell Lymphoma and Involvement of the Orbit and Ocular Adnexa

**DOI:** 10.1155/2013/581856

**Published:** 2013-04-04

**Authors:** Elham Vali Khojeini, Benjamin H. Durham, Mingyi Chen

**Affiliations:** Department of Pathology and Laboratory Medicine, School of Medicine, University of California, Davis, 4400 V Street, Sacramento, CA 95817, USA

## Abstract

Orbital and ocular adnexal lymphomas are rare and represent around 1-2% of lymphomas and about 8% of the extranodal lymphomas. However, these entities represent the majority of orbital malignancies. Lymphomas of the ocular adnexal region are primary or secondary lymphomas, and the majority of them are composed of small, mature lymphocytes, which provide a large differential diagnosis. Thus, these entities are not easily distinguished from indolent lymphoid processes such as reactive lymphoid hyperplasia. Extranodal marginal zone lymphoma is the most common lymphoma in the ocular adnexal region. However, this entity cannot be distinguished from benign lymphoid proliferations or other lymphomas composed of small, mature lymphocytes by routine histopathology. We describe a 78-year-old man who presents with bilateral upper eyelid masses, which had been present and grew in size over the past twelve months prior to his presentation. A biopsy of the mass shows a monotonous population of small, mature lymphocytes. The immunohistochemical studies performed on the eyelid mass confirmed a monoclonal proliferation of B cells expressing cyclin-D1; therefore, a final diagnosis of mantle cell lymphoma was rendered. A literature review of mantle cell lymphoma with orbital and ocular adnexal involvement and the diagnostic pitfalls in this area of hematopathology are discussed.

## 1. Introduction

The ocular adnexa comprise the tissues and structures surrounding the eye and include the following entities: the conjunctiva, eyelids, lacrimal gland, and orbital soft tissues. Orbital and ocular adnexal lymphomas are indeed rare and represent around 1-2 percent of lymphomas and about 8 percent of the extranodal lymphomas. However, these entities represent the majority of orbital malignancies [1–3]. Ocular adnexal lymphomas are primary or secondary lymphomas occurring in the orbit or ocular adnexa. The majority of lymphomas occurring in the ocular adnexa are B-cell non-Hodgkin lymphomas. The predominant subtypes of non-Hodgkin lymphoma involving this region are low-grade B-cell lymphomas, of which the majority are extranodal marginal zone lymphomas [1, 4]. Less common B-cell lymphoma subtypes include follicular lymphoma, diffuse large B-cell lymphoma, plasmacytoma, lymphoplasmacytic lymphoma, mantle-cell lymphoma, and even hairy cell leukemia [1, 4]. However, the distinction of benign and malignant lymphoid proliferations of the ocular adnexa cannot be achieved by clinical or radiological criteria and requires the concerted efforts of histological, immunophenotypical, and molecular analyses as well [1]. 

Mantle cell lymphoma (MCL) is an uncommon type of B-cell lymphoma that represents 3–10 percent of the non-Hodgkin mature B-cell lymphomas [5]. MCL predominantly occurs in older patients with a higher frequency in men, usually presenting with widespread disease, and has an aggressive clinical course. Mantle cell lymphoma most commonly occurs in lymph nodes; however, it can also be rarely present in extranodal sites such as the orbit and ocular adnexal structures, which have been reported to comprise about 6 percent of the lymphomas in these areas. There are only a few studies with a limited number of cases of MCL in the ocular adnexa described in the literature [5].

We describe a case of a 78-year-old male with no prior history of lymphoma who presents with bilateral upper eyelid masses that are diagnosed as mantle cell lymphoma involving the ocular adnexa, which is a rare cause of orbital and ocular adnexal lymphoma. We also review the literature in this area of hematopathology with emphasis placed upon the differential diagnosis and some of the diagnostic pitfalls that can be encountered.

## 2. Case Presentation

**Figure 1:**
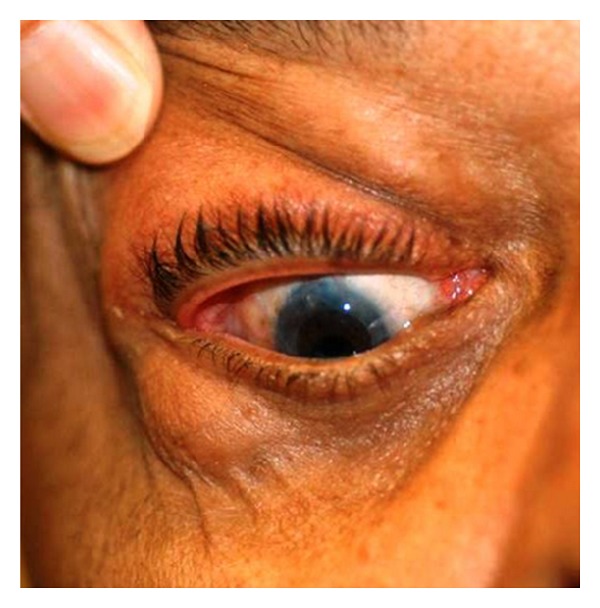
“Salmon pink,” painful masses of the right and left orbits located in the temporal aspects of the anterior orbits, which had been growing over the past twelve months.

**Figure 2:**
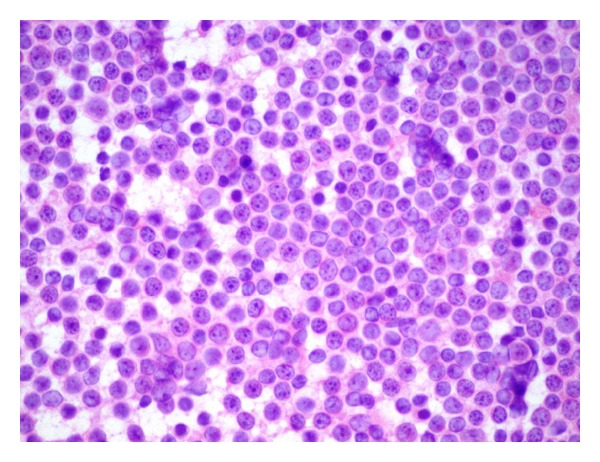
Touch imprint of the eyelid masses showing a monotonous population of small, mature lymphocytes. (Wright-Giemsa; 500x magnification).

We present a case of a 78-year-old man who presents with bilateral eyelid masses (Figure 1) that have been increasing in size over the past 12 months prior to his presentation. He did not experience other symptoms such as loss of vision, burning, itching, floaters, photophobia, or double vision. On examination he had bilateral, rubbery, mobile masses at the superior temporal aspects of the anterior orbits. These masses are hard and painful to touch. The masses are excised and show a yellow, lobulated tissue averaging 1.0 cm in greatest dimension. A touch imprint (Figure 2) was provided, and the remainder of the specimen is submitted for permanent section evaluation and flow immunophenotypical analysis. 

**Figure 3:**
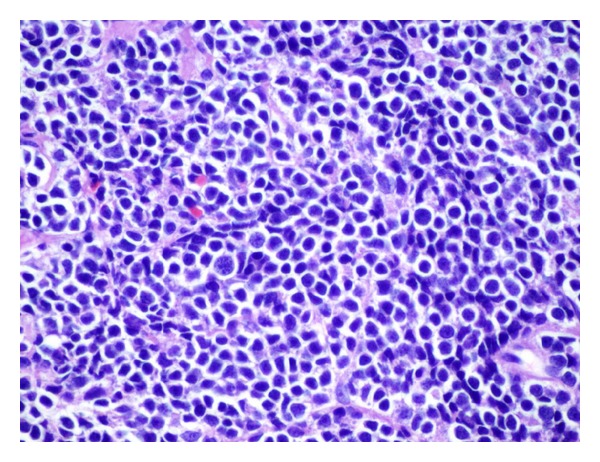
The lacrimal masses showing a monotonous proliferation of small lymphocytes with hyperchromatic, angulated nuclei and scant cytoplasm. There are hyalinized vessels noted in the background. (H&E; 400x magnification).

**Figure 4:**
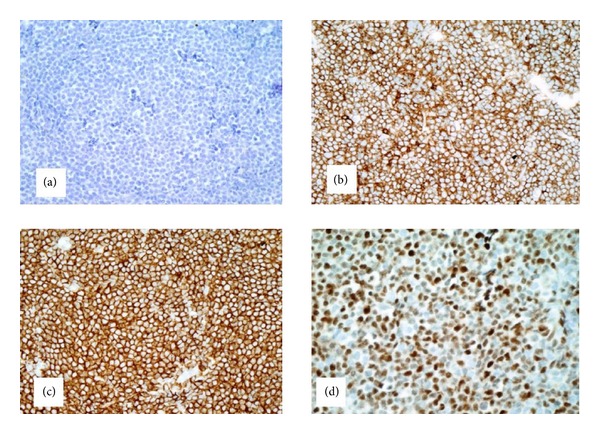
(a) Neoplastic lymphoid cells showing a lack of immunoreactivity for AE1/AE3, which assists in ruling out carcinoma (AE1/AE3; 100x magnification). (b) and (c) CD5 and CD20, respectively, demonstrate that the cell lineage for the neoplastic lymphocytes is the B lymphocyte with aberrant coimmunoreactivity with CD5. (CD5; CD20; 100x magnification). (d) Cyclin D1 staining positivity in the neoplastic cells is strongly associated with mantle cell lymphoma (Cyclin D1; 100x magnification).

The immunophenotypical analysis shows that the lymphocyte gate comprises 29% of the cells analyzed with T cells comprising 62 percent of the flow cytometry events with the B cells comprising approximately 15 percent of the events in this region. Most of these cells coexpress CD19 and CD5. These cells are also positive for FMC-7 (14 percent) and demonstrate lambda light chain restriction with a kappa-to-lambda ratio of 1 : 13. The permanent sections (Figure 3) reveal a monotonous population of small, mature lymphocytes with angulated, hyperchromatic nuclei and scant cytoplasm within a background of hyalinized vessels. Immunohistochemical studies show that the neoplastic cells are expressing Bcl-2, CD5, CD20, CD43, and cyclin-D1. They are negative for AE1/AE3, CD3, CD10, CD23, and CD30 (Figure 4). Ki-67 is performed for evaluation of the mitotic index, which highlights 20–30 percent of the neoplastic cells. Conventional chromosome analysis of the bone marrow is performed by the standard cytogenetics protocols for neoplastic studies. Six cells were available for analysis with a karyotype of 46, XY, t(11; 14) (q13; q32) and del (11) (q21; q23). 

## 3. Discussion

The ocular adnexa comprise the conjunctiva, eyelids, lacrimal gland, and orbital soft tissues. Orbital and ocular adnexal lymphomas are indeed rare and represent around 1-2 percent of lymphomas and about 8 percent of the extranodal lymphomas. However, these entities represent the majority of orbital malignancies [1–3]. The predominant non-Hodgkin lymphoma subtype involving the orbit and ocular adnexa is extranodal marginal zone lymphoma [1, 2]. Less common B-cell lymphoma subtypes include follicular lymphoma, diffuse large B-cell lymphoma, plasmacytoma, lymphoplasmacytic lymphoma, mantle cell lymphoma, and even hairy cell leukemia [1, 4]. According to a study on ocular adnexal lymphomas by Ferry et al., around two-thirds of the patients with the less common B-cell adnexal lymphoma subtypes already have previously documented lymphoma, and the remaining one-third that involve the ocular adnexa at initial presentation almost always have widespread disease by staging [2].

In general, mantle cell lymphoma (MCL) is an uncommon type of B-cell lymphoma that represents 3–10 percent of the non-Hodgkin mature B-cell lymphomas [5]. MCL was described around four decades ago and was known at that time as centrocytic lymphoma [6]. MCL typically occurs in older individuals (median age of 60 years) with a male predominance (2 : 1). Familial forms are rare but have also been reported. In two studies by Looi et al. and Rasmussen et al., the frequency of MCL involving the ocular adnexa in these study populations was more common in men (approximately 6 : 1) with a higher male-to-female ratio than systemic mantle cell lymphoma. It is also presented more commonly in older individuals, as in mantle cell lymphoma involving the lymph nodes [6, 7]. Also, Rasmussen et al. demonstrate that of the ocular adnexal sites, mantle cell lymphoma is more commonly going to involve the orbit and eyelid. Furthermore, the Rasmussen et al. study shows bilateral disease in patients with primary ocular adnexal mantle cell lymphoma more often than in patients with secondary ocular adnexal mantle cell lymphoma. Also, patients with primary ocular adnexal mantle cell lymphoma have a shorter overall survival than patients with secondary ocular adnexal mantle cell lymphoma [7].

Clinically, MCL generally presents with extensive lymphadenopathy and splenomegaly. It can show pancytopenia or present with a leukemic picture with leukocytosis [8, 9]. MCL usually presents with stage IV disease with bone marrow involvement. In the rare cases when MCL involves the orbit and ocular adnexa, it presents with an orbital mass. The patient may or may not have proptosis, diplopia, conjunctival (salmon-pink) swelling, conjunctival redness and irritation, and ptosis caused by involvement of the dermis or the orbicularis muscle of the superior eyelid [5]. 

Morphologically, MCL is comprised of monomorphic, small-to-medium-sized lymphoid cells with irregular nuclear contours. These cells proliferate in a diffuse-to-vaguely nodular pattern. As seen in this case report, hyalinized vessels are commonly seen as well [5, 7]. Cytologically, MCL is divided into four subgroups or variants: small cell, marginal zone-like, pleomorphic, and blastoid. The small cell variant has small, round lymphocytes with clumped chromatin that mimics small lymphocytic lymphoma. In the marginal zone-like variant, the lymphocytes can have abundant pale cytoplasm resembling marginal zone lymphoma or monocytoid B cells. The pleomorphic variant is comprised of pleomorphic cells with oval-to-irregular nuclear contours with pale cytoplasm. The lymphocytes in the blastoid variant resemble lymphoblasts with dispersed chromatin and a high mitotic rate (approximately 20–30/high powered field (hpf)). The pleomorphic and blastoid variants have a more aggressive clinical course when compared to the other variants [9, 10].

MCL in the ocular region presents with the same immunohistochemical markers as in nodal mantle cell lymphoma. The neoplastic B cells typically express CD19, CD20, CD22, CD5, FMC-7, surface immunoglobin (IgM and IgD), CD43, Bcl-2, and Bcl-1 (cyclin D1). Also, as is seen in nodal mantle cell lymphoma, MCL in the orbit and ocular adnexal regions tends to be more commonly lambda restricted than kappa restricted. It is typically negative for CD23; however, it can weakly express CD23, CD11c, CD10, and Bcl-6 [10]. High expression of the proliferation index by Ki-67, P53 mutation, and deletion of P16 are associated with a more aggressive form such as the blastoid variant of mantle cell lymphoma [8].

MCL is very much a genetically driven disease process. The commonly associated translocation in the neoplastic mantle cells is t(11; 14), which results in the fusion of the CCND1 gene on chromosome 11 to the immunoglobin heavy chain locus on chromosome 14 leading to the overexpression of cyclin D1 mRNA and protein [11]. This translocation occurs at the pre-B-cell stage and is considered to be the first step of the lymphomagenesis of mantle cell lymphoma [12]. It has been suggested that cyclin D1 overexpression is not sufficient to cause overt MCL, and secondary chromosomal abnormalities are required to lead to MCL development. Some of these chromosomal abnormalities include gains and losses of the following chromosomes: gains of 3q (31–50%), 7q (16–34%), or 8q (16–36%); and losses of 1p (29–52%), 6q (23–38%), 8p, 9p (18–31%), 9q, 11q (21–59%), 13q (22–55%), or 17p (21–45%) [13, 14]; and trisomy 12 (25%) [15]. The secondary genetic changes result in further disturbance of the cell cycle and the affected DNA repair pathways. As expected, the degree of karyotypic complexity is negatively associated with patient survival [15].

The distinction of benign and malignant lymphoid proliferations of the orbit and ocular adnexa cannot be achieved by clinical or radiological criteria alone and requires the concerted efforts of histological, immunophenotypical, and molecular analyses as well [1]. Of the malignant lymphoid proliferations, extranodal marginal zone lymphoma (EMZL) rises to the top of the list for lymphomas of the orbital and ocular adnexa because this subtype comprises approximately 80–90 percent of the lymphomas in these areas [16]. EMZL is a B-cell lymphoma characterized by an expansion of a heterogeneous cell population consisting of centrocyte-like, monocytoid, and plasmacytoid tumor cells with occasional blasts in the marginal zone surrounding reactive follicles. Immunophenotypically, EMZL is positive for CD79a, CD20, CD43 (usually), and IgM. It is typically negative for BCL-2, IgD, CD10, CD23, CD5, and cyclin D1 [17]. In particular, it is usually negative for CD5, which helps differentiate EMZL from MCL and chronic lymphocytic leukemia. Furthermore, EMZLs are negative for CD10 and BCL-6, for which follicular lymphoma is usually positive [18]. 

However, CD5 and cyclin D1 are two markers that can lead to diagnostic pitfalls that could drastically impact patient care within the realm of orbital and ocular adnexal lymphomas. For example, Tasaki et al. describe two cases of CD5-positive B-cell lymphomas of ocular adnexal origin where both cases are composed of small-to-medium-sized lymphocytes that were CD5-positive, CD23-negative, and cyclin D1-negative. This caused great confusion among the evaluators because the overexpression of cyclin D1 that is associated with t(11;14) is considered a hallmark feature of MCL; however, specimen fixation and processing can drastically affect cyclin D1 expression. Thus, the evaluators performed interphase fluorescence *in situ* hybridization (FISH) and found that there was no cyclin D1 expression. Therefore, these two cases are a rare presentation of CD5-positive extranodal marginal zone lymphomas [19]. Furthermore, there can be CD5-negative mantle cell lymphomas and cyclin D1-negative (BCL1-negative) mantle cell lymphomas too. In the Looi et al. study on ocular MCL, CD5 was negative in thirty percent of the cases, which is less often seen in patients with nodal MCL. In these cases, fluorescence *in situ* hybridization is recommended to differentiate MCL from other forms of lymphoma [6]. 

There are rare forms of mantle cell lymphoma that are negative for cyclin D1 that have indistinguishable features from conventional MCL. Therefore, this form of MCL lacks the (11; 14) translocation [20, 21]. According to Gesk et al., these cases can have a high expression of cyclin D2 and cyclin D3, and the cases in Gesk et al. had the karyotype of 48, XX, t(2;12)(p12; p13), +3, +21 where the t(2;12) caused breaks in the *IGK* and *CCND2* loci on chromosomes 2 and 12, respectively, leading to an *IGK-CCND2 *fusion [22]. Also, a new marker now commonly used to help differentiate MCL from other lymphoma subtypes is SOX 11 [23], which is expressed in approximately 90% of MCLs. The Mozos et al. study shows that this marker is also positive in all studied cyclin D1-negative (Bcl-1-negative) MCLs as well and helps to distinguish this lymphoma, in conjunction with the morphology, from other types of lymphoma in the differential diagnosis such as EMZL and follicular lymphoma [23].

In addition to the low-grade B-cell non-Hodgkin lymphomas and mantle cell lymphoma, other types of lymphoma also enter into the differential diagnosis. However, non-B cell lymphomas of the ocular adnexal structures are not common and represent approximately 1–3% of all lymphomas in these sites. The majority of these tumors are due to systemic involvement by the T-cell lymphoma or extension of mycosis fungoides to these sites. Hodgkin lymphoma is exceptionally rare in this area, and there have only been a few cases ever reported [1]. 

Historically, since the clinically indolent extranodal marginal zone lymphoma is the most common type of orbital and ocular adnexal lymphoma, most treating physicians considered EMZLs to have a low rate of systemic involvement with only localized disease. Thus, the gold standard of treatment of orbital and ocular adnexal lymphoma at that time was mainly with irradiation [24]. However, mantle cell lymphoma, although rare in the orbit and ocular adnexal regions, is much more aggressive. Thus, the current management of orbital and ocular adnexal MCL, which is usually presented at an advanced stage, has been limited to palliative therapy mainly in the form of chemotherapeutic regimens. Some common treatment regimens are cyclophosphamide, vincristine, prednisone; cyclophosphamide, hydroxyl-daunorubicin, vincristine, prednisone; or mitoxantrone, chlorambucil, and prednisone. A combined immunochemotherapeutic strategy (chemotherapy plus rituximab) has been shown to increase response rates, but progression free survival (PFS) and overall survival (OS) figures are still being evaluated [24].

The overall prognosis for patients with MCL of the orbit and ocular adnexa is generally poor. In one study, twelve patients (86%) had orbital and adnexal regions MCL as the first presenting sign. The study population experienced a relapse or progression of disease within a median of 27.5 months during a follow-up period ranging from 1 to 62 months. Eight of the patients (67%) had their relapse in the orbital and adnexal regions, and four (50%) had bilateral involvement [7]. In another study on patients with ocular MCL, almost all study patients had a clinical course marked by recurrent disease. The median progression free survival (PFS) was 12 months, and the median overall survival (OS) was 57 months. The International Prognostic Index was high, intermediate, or low intermediate for 7 out of 8 cases, indicating a predicted 5-year survival rate of 43 percent to 51 percent for most of the cases [4]. Furthermore, Rasmussen et al. discuss that there is also a significant difference in survival between patients with MCL who first present with orbital and adnexal regions MCL compared to patients with a previous diagnosis of MCL who develop secondary orbital and ocular adnexal MCL. The overall survival was 43 months for those first presenting with ocular adnexal MCL while it was 51 months for those with secondary ocular adnexal MCL. Furthermore, bone marrow involvement was seen in 79% of those first presenting with ocular adnexal MCL as opposed to 57% in those with secondary ocular adnexal MCL [7].

 This case report of a 78-year-old man presenting with bilateral eyelid masses and no prior history of lymphoma is a nice clinical example of how diagnostic zebras do occur and can greatly impact the clinical management of a patient. Therefore, this case emphasizes the importance of utilizing the concerted efforts of clinical and radiological criteria with histological, immunophenotypical, and genomic analyses to achieve the correct diagnosis for proper patient care [1]. Thus, entities such as mantle cell lymphoma (MCL) of the orbit and ocular adnexa do occur and are much more clinically aggressive than the more common entity of extranodal marginal zone lymphoma (EMZL) that occurs in the orbital and ocular adnexal regions [16]. Although this case has a rare but straight-forward diagnosis of MCL involving the orbit and adnexal regions, one must also remember the discussion about how CD5-positive EMZL, CD5-negative MCL, and cyclin D1-negative MCL do indeed exist and can cause diagnostic pitfalls. Thus, the standard of care in patients with orbital and ocular adnexal lymphomas, as well as other hematopoietic entities, requires a thorough workup with the integration of clinical, histological, immunophenotypical, and genomic data in every case because one never knows what diagnostic rarity will surface.

## References

[1] Coupland SE, Krause L, Delecluse HJ (1998). Lymphoproliferative lesions of the ocular adnexa: analysis of 112 cases. *Ophthalmology*.

[2] Ferry JA, Fung CY, Zukerberg L (2007). Lymphoma of the ocular adnexa: a study of 353 cases. *American Journal of Surgical Pathology*.

[3] Rootman DB, Mavrikakis I, Connors JM, Rootman J (2011). Primary, unilateral ocular adnexal lymphoma: disease progression and long-term survival. *Ophthalmic Plastic & Reconstructive Surgery*.

[4] Nola M, Lukenda A, Bollmann M, Kalauz M, Petrovečki M, Bollmann R (2004). Outcome and prognostic factors in ocular adnexal lymphoma. *Croatian Medical Journal*.

[5] Seregard S, Milton Boniuk E Ocular Adnexal Lymphomas Five Case Presentations.

[6] Looi A, Gascoyne RD, Chhanabhai M, Connors JM, Rootman J, White VA (2005). Mantle cell lymphoma in the ocular adnexal region. *Ophthalmology*.

[7] Rasmussen P, Sjö LD, Prause JU, Ralfkiaer E, Heegaard S (2009). Mantle cell lymphoma in the orbital and adnexal region. *British Journal of Ophthalmology*.

[8] Vose JM (2012). Mantle cell lymphoma: 2012 update on diagnosis, risk-stratification, and clinical management. *American Journal of Hematology*.

[9] Swerdlow SH, Campo E, Seto M, Muller-Hermelink HK, Swerdlow SH, Campo E, Harris NL (2008). Mantle cell lymphoma. *WHO Classification of Tumours of Haematopoietic and Lymphoid Tissues*.

[10] Sander B (2011). Mantle cell lymphoma: recent insights into pathogenesis, clinical variability, and new diagnostic markers. *Seminars in Diagnostic Pathology*.

[11] Hon C, Chan RTT, Ma ESK, Shek TW, Yau K, Au WY (2006). Lymphomatous proptosis as a novel feature of mantle cell lymphoma. *Leukemia and Lymphoma*.

[12] Li JY, Gaillard F, Moreau A (1999). Detection of translocation t(11;14)(q13;q32) in mantle cell lymphoma by fluorescence in situ hybridization. *American Journal of Pathology*.

[13] Rubio-Moscardo F, Climent J, Siebert R (2005). Mantle-cell lymphoma genotypes identified with CGH to BAC microarrays define a leukemic subgroup of disease and predict patient outcome. *Blood*.

[14] Beà S, Ribas M, Hernández JM (1999). Increased number of chromosomal imbalances and high-level DNA amplifications in mantle cell lymphoma are associated with blastoid variants. *Blood*.

[15] Cuneo A, Bigoni R, Rigolin GM (1999). Cytogenetic profile of lymphoma of follicle mantle lineage: correlation with clinicobiologic features. *Blood*.

[16] Yoo SB, Kim YA, Jeon YK, Kim CW (2008). CD5-undetected by immunohistochemistry, t(11;14)(q13;q32)-positive conjunctival mantle cell lymphoma: a case report. *Pathology Research and Practice*.

[17] Campo E, Miquel R, Krenacs L, Sorbara L, Raffeld M, Jaffe ES (1999). Primary nodal marginal zone lymphomas of splenic and MALT type. *American Journal of Surgical Pathology*.

[18] Jakobiec FA (2008). Ocular adnexal lymphoid tumors: progress in need of clarification. *American Journal of Ophthalmology*.

[19] Tasaki K, Shichishima A, Furuta M, Yoshida S, Nakamura N, Abe M (2007). CD5-positive mucosa-associated lymphoid tissue (MALT) lymphoma of ocular adnexal origin: usefulness of fluorescence in situ hybridization for distinction between mantle cell lymphoma and MALT lymphoma. *Pathology International*.

[20] Fu K, Weisenburger DD, Greiner TC (2005). Cyclin D1-negative mantle cell lymphoma: a clinicopathologic study based on gene expression profiling. *Blood*.

[21] Rosenwald A, Wright G, Wiestner A (2003). The proliferation gene expression signature is a quantitative integrator of oncogenic events that predicts survival in mantle cell lymphoma. *Cancer Cell*.

[22] Gesk S, Klapper W, Martín-Subero JI (2006). A chromosomal translocation in cyclin D1-negative/cyclin D2-positive mantle cell lymphoma fuses the CCND2 gene to the IGK locus. *Blood*.

[23] Mozos A, Royo C, Hartmann E (2009). SOX11 expression is highly specific for mantle cell lymphoma and identifies the cyclin D1-negative subtype. *Haematologica*.

[24] Hatef E, Roberts D, McLaughlin P, Pro B, Esmaeli B (2007). Prevalence and nature of systemic involvement and stage at initial examination in patients with orbital and ocular adnexal lymphoma. *Archives of Ophthalmology*.

